# Chronic sequelae of foodborne disease.

**DOI:** 10.3201/eid0304.970405

**Published:** 1997

**Authors:** J A Lindsay

**Affiliations:** Food Science and Human Nutrition Department, University of Florida, Gainesville 32611, USA. jali@gnv.ifas.ufl.edu

## Abstract

In the past decade the complexity of foodborne pathogens, as well as their adaptability and ability to cause acute illness, and in some cases chronic (secondary) complications, have been newly appreciated. This overview examines long-term consequences of foodborne infections and intoxications to emphasize the need for more research and education.


### Chronic Sequelae of Foodborne Disease

**James A. Lindsay**
University of Florida, Gainesville, Florida, USA

In the past decade, the complexity of foodborne pathogens, as well as their adaptability and ability to cause acute illness, and in some cases chronic (secondary) complications, have been newly appreciated. This overview examines long-term consequences of foodborne infections and intoxications to emphasize the need for more research and education.

The term foodborne disease encompasses a variety of clinical and etiologic conditions and describes a subset of enteric disease (1-4), which in the United States ranks second in prevalence to respiratory disease (2). In foodborne disease, the food may act as a vehicle for the transmission of actively growing microorganisms or products of metabolism (toxins), or it may have a passive role as a vehicle for the transmission of nonreplicating bacteria, viruses or protozoa, or stable biologic toxins. In most cases, the clinical conditions usually associated with foodborne disease are acute: diarrhea, vomiting, or other gastrointestinal manifestations such as dysentery. However, other pathophysiologic responses may occur independently or accompany acute-phase responses (1-4). A number of chronic sequelae may result from foodborne infections, including ankylosing spondylitis, arthropathies, renal disease, cardiac and neurologic disorders, and nutritional and other malabsorptive disorders (incapacitating diarrhea). The evidence that microorganisms or their products are either directly or indirectly associated with these long-term sequelae ranges from convincing to circumstantial (1-4). The reason for this disparity is that, except in rare circumstances, chronic complications are unlikely to be identified or epidemiologically linked to a foodborne cause because these data are not systematically collected. Moreover, host symptoms induced by a specific pathogen or product of a pathogen are often wide-ranging and overlapping and therefore difficult to link temporally to a specific incident. These impediments manifest themselves because the problems associated with chronic disease can result from an infection without overt illness. Alternatively, the chronic sequelae may be unrelated to the acute illness and may occur even if the immune system successfully eliminates the primary infection; therefore, activation of the immune system may initiate the chronic condition as a result of an autoimmune response (2-4). The variability of the human response—from overt illness to chronic carrier status—is perhaps the most confounding issue.

Address for correspondence: James A. Lindsay, Food Science and Human Nutrition Department, University of Florida, Gainesville, FL 32611 USA; fax 352-392-9467; e-mail: jali@gnv.ifas.ufl.edu.

### Cost of Chronic Sequelae

As the incidence of foodborne disease increases, the incidence of chronic sequelae may also rise. Several authors have estimated that chronic sequelae may occur in 2% to 3% of foodborne disease cases and suggest that the long-term consequences to human health and the economy may be more detrimental than the acute disease. An estimated 80 million cases of foodborne disease occur annually in the United States, which suggests significant morbidity figures and costs to society in the billions of dollars per year (2,4).

### Infection: The Microbe/Toxin versus the Host

Several microbial pathogens are highly adapted to parasitization, exhibiting environmentally responsive and adaptive traits that allow attachment, invasion, and replication in the host (2,4). Microbial pathogenicity can be viewed solely from the perspective of the microbe; however, this would be not only unidimensional, but also wrong (2,4). A major selective force that regulates the phenotype of an infecting microbial pathogen population is the host's immune system, which is also highly adaptive, especially in discriminating self and nonself antigens (2,4). When the host-parasite relationship is examined holistically, mechanisms that successful pathogens have apparently evolved to elude the immune system include antigenic heterogeneity or variation; sequestration, either intracellularly or in certain specific host sites; molecular mimicry, through either imitation (cross-reaction) or adsorption of host protein; and direct immune stimulation and/ or suppression (2-5).

### Rheumatoid Disease

Several bacteria, including salmonellae, induce septic arthritis by hematogenous spread to the synovial space, causing inflammation. Viable organisms are recoverable from synovial fluid, and treatment usually involves antibiotic therapy. Prognosis depends on host factors and virulence of the organism; either complete resolution or permanent joint damage can occur (1-4,6,7).

*Yersinia enterocolitica, Y. pseudotuberculosis, Shigella flexneri, Sh. dysenteriae, Salmonella* spp., *Campylobacter jejuni,* and *Escherichia coli* initiate aseptic or reactive arthritis, an acute, nonpurulent joint inflammation following infection elsewhere in the body, for example the bowel. *Klebsiella pneumoniae* has been implicated, although it appears now that the bacterium is connected with fecal carriage by ankylosing spondylitis probands (4). Although a distinct clinical disease, reactive arthritis also occurs in the Reiter syndrome triad with conjunctivitis and uveitis. A subset of patients with symptoms of reactive arthritis and Reiter syndrome get ankylosing spondylitis, a rheumatoid inflammation of synovial joints and entheses within and distal to the spine (8).

The relative risk of developing these seronegative spondyloarthropathies after a gram-negative enterobacterial infection is high for persons positive for the major histocompatibility class (MHC) antigen B27 and the cross-reacting MHC B7 group. Indeed, persons who are human leukocyte antigen (HLA)-B27 positive have an 18-fold greater risk for reactive arthritis, a 37-fold greater risk for Reiter syndrome, and up to a 126-fold greater risk for ankylosing spondylitis than persons who are HLA-B27 negative and have the same enteric infections. Other genes that may be related or act in concert appear to determine which disease is acquired (2,5,7,9). These chronic complications are related to a genetically determined host risk factor in combination with an environmental trigger. No cause-and-effect relationship of enteric pathogens in ankylosing spondylitis has been established (4); however, a low but consistent incidence (0.2% to 2.4%) of reactive arthritis occurs after outbreaks of *S.* Typhimurium, *Sh. flexneri,* and *C. jejuni*. Biotypes and phage types of *Y. enterocolitica* O:3 and O:9, endemic to Scandinavia, are either highly arthritogenic or affect a more genetically predisposed population with persistent and debilitating symptoms that may last for years (2,3).

The sharing of antigenic determinants by a microbe and its host is a frequent natural occurrence, and bacterial antigens from the pathogens that directly cross-react with MHC B27 have been demonstrated (6,9). Additionally, the plasmid-mediated synthesis of bacterial B27 “modifying factor,” a protein that binds to and subsequently alters the conformation of B27, has been reported (10). In both of these models, immune recognition of the foreign antigen leads to an autoimmune anti-B27 response. Alternatively, B27 may act nonimmunologically because dissemination of bacterial antigens to infected joints stimulates a local T-cell inflammatory response. Here, B27 may act as a receptor for bacteria or antigens thereof, facilitating invasiveness from mucosal surfaces in the gut (9). Indeed, transfected B27 on the surface of mouse L cells reportedly can alter bacterial invasion capability (11).

Despite the strong familial association related to the MHC B27 gene, B27-negative persons are known to become ill, albeit less often, but with apparently equal severity, as shown by an epidemiologic investigation of rheumatoid arthritis following the 1985 *S.* Typhimurium gastroenteritis outbreak due to contaminated milk (4).

### Autoimmune Thyroid Disease

Graves disease is an autoimmune disease mediated by autoantibodies to the thyrotropin receptor (12,13). The first indication that the disease may be linked to infection was finding antibody titers to *Y. enterocolitica* serotype O:3 suggestive of molecular mimicry in a majority of patients with Graves disease. Several studies have shown that two low molecular weight envelope proteins of *Yersinia* contain epitopes cross-reactive with the thyrotropin receptor. These proteins are chromosomally encoded, exposed to the surface of the bacterium, and produced by both virulent and avirulent strains of *Yersinia* (*Y. pestis, Y. pseudotuberculosis, Y. enterocolitica*

VW+ and WV-). In addition to autoantibody, a suppressor cell dysfunction may be involved in Graves disease (12,13). Severe hypothyroidism may also result from chronic intestinal giardiasis due to infection by *Giardia lamblia*; treatment with metronidazole can result in complete elimination of the parasite and recovery of regular intestinal thyroid hormone absorption (14).

### Inflammatory Bowel Disease

Inflammatory bowel disease is the collective term for Crohn disease and ulcerative colitis. While both infections are chronic inflammatory diseases with histologic infiltrates of macrophages and lymphocytes and a prolonged clinical course, the primary clinical and pathologic effects are gastrointestinal. The infections can be difficult to differentiate because the symptoms are often similar (15). The acute clinical characteristics are diarrhea, abdominal pain, fever, and weight loss; and the acute pathologic features include a constant flux of neutrophils into inflamed mucosa, eventually penetrating the epithelium into the intestinal lumen. The chronic spontaneously relapsing disorder exhibits many of the symptoms of the acute state; however, this phase has an average symptom duration of 3.2 years before correct diagnosis. Abdominal abscesses are a common and dangerous complication of Crohn disease, while in ulcerative colitis, abdominal perforations may lead to peritonitis. Crohn disease involves the ileum or colon (anaerobes are important), while ulcerative colitis appears restricted to the colon (aerobes are important). Nationality and familial associations suggest a genetic predisposition for the disease (4,15).

Although the cause of inflammatory bowel disease and the mechanism(s) for spontaneous exacerbations and remissions are unknown, much research has focused on transmissible agents, including foodborne pathogens. An association between bacterial L-forms and inflammatory bowel disease has been sporadically reported, with isolation of *Pseudomonas*, *Mycobacterium*, *Enterococcus fecalis*, and *E. coli* from affected tissue but not from appropriate controls. There is considerable debate as to whether L-forms are pathogenic in humans or persist in affected tissue.

*Mycobacterium paratuberculosis*, the causative agent of Johne disease in ruminants, may be associated with Crohn disease through the production of L-forms of the bacterium. Subclinically infected cows shed *M. paratuberculosis*, and the organism has been identified in pasteurized milk by polymerase chain reaction specific for the *M. paratuberculosis* insertion sequence IS900. The pathogen model suggests that a susceptible human neonate first contracts the organism after ingesting commercial dairy products. This invokes an antigen-poor (lacking a cell wall) L-form that grows slowly and persists in the lamina propria, stimulating a chronic low-grade inflammation. The immune response increases in severity over years without bacterial replication, ultimately producing the pathologic features of Crohn disease (15,16). Another model proposes an autoimmune phenomenon mediated by alterations in inflammatory cytokine profiles, possibly as a result of infection (4).

Recent immunocytochemical techniques demonstrated antigens to *Listeria monocytogenes*, *E. coli*, and *Streptococcus* spp. in Crohn disease tissues. Macrophages and giant cells immunolabelled for antigen specific to these organisms were found beneath ulcers, around abscesses, along fissures, within the lamina propria, in granulomas, and in germinal centers of mesenteric lymph nodes (17).

### Superantigens and Autoimmunity

In contrast to conventional antigens, superantigens interact with the variable side of the Vß chain of the T-cell receptor by recognizing elements shared by a subset of T cells. Depending on the type of interaction, recognition can have different consequences, including proliferation and expansion, suppression (clonal deletion), or, alternatively, induction of prolonged unresponsiveness (anergy) or cell death (apoptosis) (18-21). Superantigens from several foodborne bacteria (e.g., *Staphylococcus*, *Streptococcus*, *Yersinia*, and *Clostridium*) have been isolated and characterized. Many are thought to be associated with several autoimmune disorders, for example, rheumatic heart disease, rheumatoid arthritis, multiple sclerosis, Graves disease, Sjogren syndrome, autoimmune thyroiditis, psoriasis, Kawasaki disease, Crohn disease, and insulin-dependent diabetes mellitus (6,18-24).

Although it is accepted that superantigens have a role in autoimmune disorders, the acceptance is based on extensive animal model studies (6,18-24) but limited human clinical studies. In human diseases where superantigens have been clearly demonstrated as the cause, for example, toxic shock syndrome, initial T-cell proliferation and T-cell receptor–mRNA upregulation have been observed, but the long-term sequelae in terms of T-cell function are unknown (22). Recent studies suggest that superantigens may also cause an acute flare of a disease within patients in remission from a preexisting autoimmune disorder.

### Renal Disease

After colitis caused by *E. coli* O157:H7 and other enterohemorrhagic strains of *E. coli*, hemolytic uremic syndrome develops in some patients (1,2,25). The syndrome is characterized by a triad of symptoms: acute renal failure, thrombocytopenia, and microangiopathic hemolytic anemia. Acute renal failure is the leading cause of death in children, and thrombocytopenia is the leading cause of death in adults. Hemolytic uremic syndrome is a worldwide problem that mirrors the distribution of *E. coli* O157:H7 and other Shiga and Shiga-like toxin–producing microorganisms. Outbreaks of hemorrhagic colitis and subsequent cases of hemolytic uremic syndrome have developed as a result of various food vehicles. Besides O157:H7, other Shiga-like toxin–producing *E. coli*, *Citrobacter*, *Campylobacter*, *Shigella*, *Salmonella*, and *Yersinia* have been linked to the disorder (1,2,25,26).

The toxin-mediated damage to the kidneys may not be limited to the glomerular endothelial cells as once thought but may include the tubular epithelial cells (26-28). Binding studies showed the toxins to be specific for the glycosphingolipid globotriaosylceramide (Gb3), which is present on renal but not umbilical endothelial cells. This may account for the differential sensitivity of renal cells to toxin-induced damage, since Gb3 was present in the glomeruli of infants under 2 years of age but not in the glomeruli of adults. Thus, the presence of Gb3 in the pediatric renal glomerulus may be a risk factor for development of hemolytic uremic syndrome (28). Characterization of the Shiga toxin receptor has led to a potential preventive treatment (4).

### Neural and Neuromuscular Disorders

Guillain-Barré syndrome is a subacute, acquired, inflammatory demyelinating polyradiculoneuropathy that frequently occurs after acute gastrointestinal infection. The disease is characterized by alexia, motor paralysis with mild sensory disturbances, and an acellular increase in the total protein content in the cerebrospinal fluid. The disease occurs worldwide and is the most common cause of neuromuscular paralysis. Cases have three dominant characteristics: the predilection to nerve roots, mononuclear infiltration of peripheral nerves, and eventual demyelination (primary axonal degeneration) (29). Severe cases tend to occur in the summer and have been linked to previous infection with *C. jejuni*, although other enteric pathogens may trigger the syndrome.

Some controversy exists regarding whether Guillain-Barré syndrome is an autoimmune disease. Although adequate data exist to classify the syndrome as an autoimmune disease (four major Rose-Witebsky criteria are almost completely met), the immunologic mechanisms at work in Guillain-Barré syndrome triggered by *C. jejuni* are likely to be complex (29-31). Studies of the relationship between Guillain-Barré syndrome and *C. jejuni* support the hypothesis of molecular mimicry, since peripheral nerves may share epitopes with surface antigens of *C. jejuni* (32). This has been supported by studies in which anti-GM1 IgG antibodies recognized surface epitopes on intact *C. jejuni*, and the reaction was strain-specific for certain Penner serotypes. There are inconclusive data with regard to Guillain-Barré syndrome and HLA, although some studies have shown a predilection for the HLA-B35 haplotype (29-31). Cytokines may be responsible for inducing the inflammatory process and probably play a role in the response leading to nerve demyelination. Complement has a role in the process leading to nerve damage, possibly through the production of activation products, which lead to an increase in the permeability of the blood nerve barrier, which perpetuates the inflammation. Although Guillain-Barré syndrome might be considered an autoimmune response, it also serves as an example of a disease with an infectious origin, a disease that entails the integrated actions of both humoral and cellular immunity.

Ciguatera poisoning is the most common foodborne disease related to the consumption of fin fish; this distinctive clinical syndrome is characterized by a plethora of gastrointestinal, neurologic, and sometimes cardiovascular features (33,34). Two toxins are involved in toxicosis. Ciguatoxin-1 (cig-1), the principal toxin, is a heat stable, lipid-soluble polyether that is not inactivated by heat, cold, or gastric juices, nor eliminated by drying, salting, smoking, or marinating. Cig-1 induces membrane depolarization in nerve and muscle tissue by opening voltage-dependent sodium channels. A second toxin, maitotoxin, is water-soluble and opens calcium channels. The role of this second toxin in the pathophysiology of the disease is less well understood. The acute symptoms of the toxicosis are varied and include paresthesia of the extremities, circumoral paresthesia, reversal of hot and cold sensations, dental pain, myalgias, arthralgias, generalized pruritus, cranial nerve dysfunction, and dysuria. Severe acute symptoms require urgent care with parenteral atropine for bradyarrhythmias. Mannitol is often administered to counter the effect of the toxin on the sodium channels; however, the mechanism of action is unknown, and the therapy is useless after 24 hours. Many of these symptoms may remain chronic and are often misdiagnosed as chronic fatigue syndrome, brain tumors, or multiple sclerosis. The management of the chronic symptoms is frustrating for the patient and clinician. Interventions include amitriptyline, tocaidine, or mexilitine to modulate sodium channels in conjunction with calcium channel blockers such as nifedipine. Antidepressants such as Prozac also appear to be useful. Patients with chronic symptoms frequently report waxing and waning of symptoms. Activities such as sexual intercourse and drinking alcohol significantly exacerbate expression. Some women with chronic symptoms report worsening during menses. Mood levels, weather conditions, and dietary constituents often exacerbate symptoms. Some clinicians advocate a strict diet that avoids all seafood, fish byproducts, nuts, and alcohol, and in some cases, patients are asked to abstain from sex. One distinctive feature in this toxicosis is that one episode of ciguatera poisoning does not confer immunity. In fact, it is likely to sensitize the patient to otherwise subthreshold doses of toxin (33,34).

Amnesic shellfish poisoning is caused by domic acid, a conjugate of kainic and glutamic acid (35). In small quantities domic acid has an excitatory effect, but in large amounts it is neurotoxic. The toxicosis is first characterized by gastrointestinal symptoms followed by neurologic dysfunction. Severe cases may be prolonged and chronic; sequelae include confusion with disorientation, paucity of speech, lack of response to deep pain due to blocking of receptors in the spinal cord, autonomic nervous system dysfunction, seizures, abnormal ocular movements, grimacing posture, myoclonus, loss of reflexes, and coma. Other prominent chronic sequelae include loss of visual-spacial recall and mononeuropathies without dementia, mimicking Alzheimer's disease. The toxicosis is particularly serious in the elderly, and any deaths usually occur within this population. Valium, calcium channel blockers, phenobarbital, diazapam, thiobarbiturates, and hypothermia are treatments for patients with severe and chronic cases.

### General Immunity, Organ Impairment, and Neurologic Disorders

Toxoplasmosis due to *Toxoplasma gondii* is a chronic latent parasitic infection (36,37). In humans the parasite exists in two forms: the tachyzoite, the rapidly multiplying stage that actively invades host cells and represents the principal form of the acute phase of the disease; and the bradyzoite, the form that multiplies very slowly in host cells, resulting in the formation of cysts that persist in tissues. Toxoplasma infection in humans is usually asymptomatic because of effective immunity involving antibodies, T cells, and cytokines. Activated macrophages, CD4 and CD8 lymphocytes, and the cytokines IFN-$\gamma$, TNF-$\alpha$, and IL-6 play a major role in control of both the acute infection and maintenance or prevention of the chronic stage (37,38). Indeed, treatment with IFN-$\gamma$ is used to control passage into the chronic stage, and treatment with anti-IFN-$\gamma$ reactivates chronic infection (39). The production of nitric oxide may have opposing effects. Nitric oxide production protects against *T. gondii* and at the same time limits the immune response, probably contributing to the establishment of the chronic state (40).

The incidence of congenital toxoplasmosis is uncertain but may be as high as 9,500 cases a year (1). The percentage attributable to food is uncertain; however, consumption or contact with contaminated meat is more important as a cause than is contact with cats (1). Congenital impairments associated with maternal toxoplasmosis infection passed to the fetus include hearing loss, visual impairment (retinal lesions, strabismus), and slight to severe mental retardation. These impairments are still present in 80% of persons who reach the age of 20 years (1). Chronic toxoplasmic encephalitis may occur when a person's immune system is impaired. Indeed, toxoplasmic encephalitis marked by dementia and seizures has become the most commonly recognized cause of central nervous system opportunistic infections in AIDS patients. Additionally, it appears that certain cancer treatments weaken the immune system, and old infections in the muscles can become reactivated, causing severe complications or death (1,41,42).

Helminth parasites can cause serious disease in infected persons (42). The impact of helminth infections is due less to the severity of the diseases they cause, than to the vast number of persons infected. For example, more than one billion people are infected with the largest intestinal nematode, *Ascaris lumbricoides*. Although there is usually no overt clinical sign of infection, disease can arise from an overwhelming infection or an inappropriate immune response. Additionally, infected persons frequently harbor more than one parasite for years. Most intestinal helminth parasites have direct life cycles, with no intermediate host or vector, and are transferred by contaminated food. Some species, such as *Trichuris* (whipworm) and *Enterobius* (pinworm), are restricted to the gut, but others, such as *Ascaris*, have tissue-migrating phases. All, however, induce a dramatic expansion of the Th2 lymphocyte subset. It remains unclear whether these Th2-derived responses (induction of interleukin-4 [IL-4] and down-regulation of IFN-$\gamma$), resulting in stimulation of IgG1 and IgE isotypes, eosinophilia, and mastocytosis are responsible for the immune-mediated pathologic response. Immunologic lesions may occur where early infection is associated with a strong T-cell proliferative response that becomes down-regulated in established chronic disease (evidence of a Th1 defect in the chronic disease). In ascariasis, an allergic response generated by the lung migratory phase (chronic immune sensitization) can cause pneumonia and, in animal models, spontaneous development of idiopathic bronchial asthma. A formative influence on the response of the immune system is the antigenic environment during pregnancy. Children born to infected mothers may have significantly higher susceptibility to the same infection later in life (42).

Viral agents induce autoimmune disorders, and one potential mechanism of induction is molecular mimicry (43,44). Hepatitis A virus infection is a well-recognized cause of acute hepatitis with jaundice in adults. In most affected persons, the course is usually relatively short-lived and benign, and symptoms are usually resolved within weeks. Occasionally, relapses occur after initial recovery, or recovery is marked by severe or prolonged cholestasis. However, even in these cases recovery is usual. Chronic sequelae of hepatitis A virus infections are rare and poorly defined; however, several recent studies suggest that hepatitis A virus infection triggers the onset of (idiopathic) autoimmune chronic active hepatitis within a genetically predisposed subgroup. Apparently, the chronic disorder may develop despite normal serologic response to hepatitis A virus infection. The triggering factors and mechanism of action remain ill-defined; however, in the most recent study, the authors concluded that hepatitis A virus infection may be the precipitating event in the pathogenesis of this disorder (45).

Metabolic activation and detoxification play a crucial role in determining the toxic response of humans to mycotoxin exposure. These highly toxic secondary metabolites are produced by a wide variety of molds including *Aspergillus*, *Fusarium*, and *Penicillium*. Mycotoxins exhibit properties of acute, subacute, and chronic toxicities with some molecules being carcinogenic, mutagenic, and teratogenic. Because mycotoxins are resistant to food processing and do not degrade at high temperatures, they enter the human food supply (46-49).

In many cases, the relationship between mycotoxins as the causative agent of disease in humans is difficult to determine. Acute effects of gastroenteritis may be easily identified; however, chronic effects often result from ingestion of low to moderate levels and can be difficult to recognize (46-50). The most threatening effects of ochratoxin A are its nephrotoxicity and carcinogenicity. Ochratoxin A is increasingly involved in an endemic nephropathy, a human chronic interstitial neuropathy that is usually associated with urinary tract tumors. Aflatoxins have been implicated in both acute and chronic liver disease in humans; however, other organs (kidney, spleen, pancreas) may also be affected (51). The best studied chronic effects are those induced by the fumonisins, zearalenone, and trichothecene mycotoxins produced by *Fusarium* sp. (49). Fumonisin levels in corn-based foods have been statistically associated with an increased risk of human esophageal cancer. Zearalenone is an estrogenic mycotoxin. Ingestion by animals, especially swine, causes hyperestrogenism with symptoms of enlargement and prolapse of the uterus, atrophy of ovaries and testicles, enlargement of mammary glands, and infertility. This mycotoxin might add to the estrogen load of humans. Human consumption of trichothecene-contaminated foods causes acute symptoms of headaches, chills, severe nausea, vomiting, and visual disturbances, which may last 7 to 10 days. Since trichothecenes modulate immune function, over time mycotoxicosis could reduce immune resistance to infectious diseases, facilitate tumor growth through reduced immune function, and cause autoimmune disease (48).

### Heart and Vascular Diseases

Several foodborne pathogens have been either directly or indirectly associated with endocarditis and myocarditis, and any heart damage appears to be permanent (2,52). Persons with ankylosing spondylitis linked to enteric pathogens as the trigger show a high incidence of cardiac conduction abnormalities, which may be sequelae to other seronegative arthropathies. A possible connection between foodborne gram-negative bacteria and atherosclerosis has been proposed, suggesting that the bacteria gain access to the lymphoid and general circulation with relative frequency. Endotoxin from degrading bacteria in macrophages may act in concert with the inflammatory factors (cytokines) induced by endotoxin from endothelium and smooth muscle cells. Although the process of atherogenesis is complex and involves many factors, the hypothesis is attractive and provides a model system for further study. Oxidative stress responses by *E. coli* and *S*. Typhimurium and the induction of the peroxide stimulon and the superoxide stimulon have also been recently implicated in atherosclerosis, rheumatoid arthritis, and inflammatory bowel disease (53).

### Nutritional and Gastrointestinal Disturbances

Enteric pathogen-induced diarrhea may lead to a variety of conditions including loss of fluids, anorexia, and malabsorption of nutrients, all forms of malnutrition. The enteric pathogens that cause malabsorption and nutrient loss vary and include *Enterobacteriaceae*, Rotavirus, *Amoeba, Cryptosporidium*, and *Giardia* (2,54-58). Unless treated with antimicrobial drugs, many diarrheal episodes become chronic; however, the stress of even short periods of diarrhea may result in subtle changes in immunologic status. The extent of the diseases depends mostly on the immune status of the person and may last for several years or for life. Death due to diarrheal illness in the immunosuppressed and in persons with AIDS is nearly 80%. No effective treatment is available, although treatment with several antibiotics in combination shows promising results. However, AIDS patients may also develop further sequelae. *Cryptosporidium* are host-adapted, which may lead to pulmonary or tracheal cryptosporidiosis accompanied by coughing and frequent low-grade fever. In these cases there is no effective treatment. Similarly, in cyclosporidiosis, AIDS patients' enteric infection is chronic. Long-term prophylaxis with trimoxazole is required, and discontinuation of the treatment causes severe relapse (2,54-58).

Severe cases of diarrhea lasting months or years and characterized by dysentery, with foul-smelling, mucous bloody stools accompanied by flatulence and abdominal distention, may result from *C. jejuni*, *Citrobacter*, *Enterobacter*, or *Klebsiella* enteric infections. These infections always require extensive antibiotic therapy and usually result in failure to thrive. Enteric infections may alter bowel permeability which allows absorption of otherwise excluded food components. Proteins that can modulate the immune system can be absorbed possibly with deleterious consequences such as the induction of autoimmunity and atopy. Several studies of both human and porcine models indicate that significant quantities of unwanted proteins can be absorbed by damaged gut tissue and that maximum expression of diarrhea corresponds with peak protein uptake (2).

*Helicobacter pylori* is the undisputed cause of chronic gastritis. Environmental sources indicate that *H. pylori* can survive in water, chilled foods, milk, and fresh vegetables for several days because of fecal contamination. The species has never been isolated from these sources; however, infectious, viable but nonculturable (nonspiral coccoid) bodies may survive in fresh water for more than a year. *H. pylori* can be found in human feces and can be transmitted directly from person to person by the fecal-oral or oral-oral route. *H. pylori* can be found in several animal reservoirs; however, the possibility of animal-to-animal or zoonotic transmission is unknown (59,60). Ingestion of *H. pylori* leads to acute gastritis, and colonization of the stomach is virtually always accompanied by chronic inflammation that disappears within 6 to 12 months after eradication of the infection. Infections are generally acquired during childhood or adolescence and result in chronic gastritis lasting for decades or life. On the basis of histologic and serologic follow-up studies, this chronic gastritis has been suggested as an important risk factor (odds ratio 9.0; $p = 0.001$) or first stage in a multistep process leading to gastric mucosal atrophy, intestinal metaplasia, and eventually gastric cancer (61,62).

### Chronic Sequelae and Personality Changes

One area that has received scant interest is the effect of a chronic infection on human personality factors. Personality changes might be predicted: continual pain from arthritis, an irritable bowel, or chronic diarrhea would be enough to make anyone temperamental, moody, or depressed. Studies using Cattell's 16 Personality Factor questionnaire showed highly significant correlations ($p < 0.01 - 0.002$) between chronic toxoplasmosis and several personality factors (63,64). Men and women showed distinct differences in behavioral states. For men, low superego strength, protension, guilt-proneness, and group dependency were positively associated, whereas in women the related factors were affectothymia, alexia, untroubled adequacy, and self-sufficiency. A correlation of the intensity of the personality factor-shifts with the duration of the infection suggested that the infection per se induced the shift in personality, not vice versa.

An exploratory study using the 16 Personality Factor questionnaire and the Holmes and Rahe Social Readjustment Rating Scale, which measures stressful life events, was made of patients with rheumatoid arthritis (65). As a group, patients with rheumatoid arthritis exhibited higher stress at disease onset ($p < 0.01$); a large high-stress-at-onset subgroup of rheumatoid arthritis patients had a worse prognosis. Although there were important personality changes in the high-stress-at-onset-rheumatoid arthritis patients, the study concluded that the interaction between the variables that determined personality changes were very complex and could not simply be referred to as the "rheumatoid arthritis personality" complex.

### Conclusion

Foodborne diseases are for the most part preventable; however, there is an inherent risk associated with the consumption of certain types of uncooked foods. Recognition by the public health community and the public that many foodborne illnesses may have serious chronic sequelae would help eliminate many illnesses and reduce health-care cost. Public health authorities could make a substantial impact by reducing poor or unhygienic food production or food-handling practices and by educating the public about how harmful microorganisms enter the food chain and how they can be avoided.

### References

1. Foegeding PM. Foodborne pathogens: risks and consequences. Council for Agricultural Science and Technology. Task Force Report 1221; 1994.
2. Archer DL, Young FE. Contemporary issues: diseases with a food vector. Clin Microbiol Rev 1988;1:377-98.
3. Bunning VK. Immunopathogenic aspects of foodborne microbial disease. Food Microbiology 1994;11:89-95.
4. Bunning VK, Lindsay JA, Archer DL. Chronic health effects of foodborne microbial disease. World Health Stat Quart 1997;50:51-6.
5. Miller FW. Genetics of autoimmune diseases. Exp Clin Immunogenet 1995;12:182-90.
6. Behar SM, Porcelli SA. Mechanisms of autoimmune disease induction: the role of the immune response to microbial pathogens. Arthritis Rheum 1995;38:458-76.
7. Yu DT, Thompson GT. Clinical, epidemiological and pathogenic aspects of reactive arthritis. Food Microbiology 1994;11:97-108.
8. Sieper J, Braun J. Pathogenesis of spondylarthropathies. Arthritis Rheum 1995;38:1547-54.
9. Gaston JH. How does HLA-B27 confer susceptibility to inflammatory arthritis? Clin Exp Immunol 1990;82:1-2.
10. McGuigan LE, Prendergast JK, Geczy AF, Edmonds JP, Bashir HV. Significance on non-pathogenic cross-reactive bowel flora in patients with anklosing spondylitis. Ann Rheum Dis 1986;45:566-71.
11. Kapasi K, Inman RD. HLA-B27 expression modulates Gram-negative bacterial invasion into transfected L-cells. J Immunol 1992;148:3554-9.
12. Wolf MW, Misaki T, Bech K, Tvede M, Silva JE, Ingbar SH. Immunoglobulins of patients recovering from *Yersinia enterocolitica* infections exhibit Graves' disease-like activity in human thyroid membranes. Thyroid 1991;1:315-20.
13. Luo G, Seetharamaiah GS, Niesel DW, Zhang H, Peterson JW, Prabhakar BS, Klimpel GR. Purification and characterization of *Yersinia enterocolitica* envelope proteins which induce antibodies that react with human thyrotropin receptor. J Immunol 1994;152:2555-61.
14. Seppel T, Rose F, Schlaghecke R. Chronic intestinal giardiasis with isolated levothyroxine malabsorption as reason for severe hypothyroidism: implications for localization of thyroid hormone absorption in the gut. Exp Clin Endocrinol Diabetes 1996;104:180-2.

15. Chiodini RJ. Crohn's disease and the mycobacteriosis: a review and comparison of two disease entities. Clin Microbiol Rev 1989;2:90-117.
16. Millar D, Ford J, Sanderson J, Withey S, Tizard M, Doran T, Hermon-Taylor J. IS900 PCR to detect *Mycobacterium paratuberculosis* in retail supplies of whole pasteurized cow's milk in England and Wales. Appl Environ Microbiol 1996;62:3446-52.
17. Liu Y, van Kruiningen HJ, West AB, Cartun RW, Cortot A, Colombel JF. Immunocytochemical evidence of *Listeria, Escherichia coli*, and *Streptococcus* antigens in Crohn's disease. Gastroenterology 1995;108:1396-401.
18. Goodacre JA, Brownlee CED, Ross DA. Bacterial superantigens in autoimmune arthritis. Br J Rheumatol 1994;33:413-9.
19. Kotb M. Bacterial pyrogenic exotoxins as superantigens. Clin Microbiol Rev 1995;8:411-26.
20. Kotb M. Infection and autoimmunity: a story of the host, the pathogen and the copathogen. Clin Immunol Immunopathol 1995;74:10-22.
21. Johnson HM, Torres BA, Soos JM. Superantigens: structure and relevance to human disease. Proc Soc Exp Biol Med 1996;212:99-109.
22. Leung DY, Meissner HC, Fulton DR, Murray DL, Kotzin BL, Schlievert PM. Toxic shock syndrome toxin secreting *Staphylococcus aureus* in Kawasaki syndrome. Lancet 1993;342:1385-8.
23. Kay RA. The potential role of superantigens in inflammatory bowel disease. Clin Exp Immunol 1995;100:4-6.
24. Conrad B, Weidmann E, Trucco G, Rudert WA, Behboo R, Ricordi C, et al. Evidence for superantigen involvment in insulin dependent diabetes mellitis aetiology. Nature 1994;371:351-5.
25. Tarr PI. *Escherichia coli* O157:H7: clinical, diagnostic, and epidemiological aspects of human infection. Clin Infect Dis 1995;20:1-10.
26. Tesh VL, O'Brien AD. The pathogenic mechanisms of Shiga toxin and the Shiga-like toxins. Mol Microbiol 1991;5:1817-22.
27. Pudymaitis A, Armstrong G, Lingwood CA. Verotoxin-resistant cell clones are deficient in the glycolipid globotriosylceramide: differential basis of phenotype. Arch Biochem Biophys 1991;286:448-52.
28. Lingwood CA. Verotoxin-binding in human renal sections. Nephron 1994;66:21-8.
29. Shoenfeld Y, George J, Peter JB. Guillain-Barré as an autoimmune disease. Int Arch Allergy Immunol 1996;109:318-26.
30. Peterson MC. Clinical aspects of *Campylobacter jejuni* infections in adults. West J Med 1994;161:148-52.
31. Bolton CF. The changing concepts of Guillain-Barré Syndrome. New Engl J Med 1995;333:1415-7.
32. Oomes PG, Jacobs BC, Hazenberg MP, Banffer JR, van der Meche FG. Anti-GM1 IgG antibodies and *Campylobacter* bacteria in Guillain-Barré Syndrome: evidence of molecular mimicry. Ann Neurol 1995;38:170-5.
33. Swift AE, Swift TR. Ciguatera. J Toxicol Clin Toxicol 1993;31:1-29.
34. Lange WR. Ciguatera fish poisoning. Am Fam Physican 1994;50:579-84.
35. Todd ECD. Domic acid and amnesic shellfish poisoning: a review. Journal of Food Protection 1993;56:69-83.
36. Smith JL. Foodborne toxoplasmosis. Journal of Food Safety 1991;12:17-57.
37. Derouin F. Pathology and immunological control of toxoplasmosis. Braz J Med Biol Res 1992;25:1163-9.
38. Khalil SS, Rashwan EA. Tumor necrosis factor-alpha (TNF-alpha) in human toxoplasmosis. J Egypt Soc Parisitol 1996;26:53-61.
39. Olle P, Bessieres MH, Malecaze F, Seguela JP. The evolution of ocular toxoplasmosis in anti-interferon gamma treated mice. Curr Eye Res 1996;15:701-7.
40. Hayashi S, Chan CC, Gazzinelli R, Roberge FG. Contribution of nitric oxide to the host parasite equilibrium in toxoplasmosis. J Immunol 1996;156:1476-81.
41. Shrittematter C, Lang W, Wiestler OD, Kleihues P. The changing patter of HIV associated cerebral toxoplasmosis. Acta Neuropathol (Berl) 1992;8:475-81.
42. Allen JE, Maizels JM. Immunology of human helminth infection. Int Arch Allergy Immunol 1996;109:3-10.
43. Moyer L, Warwick M. Mahoney FJ. Prevention of hepatitis A virus infection. Am Fam Physician 1996;54:107-14.
44. von Herrath MG, Oldstone MBA. Virus-induced autoimmune disease. Curr Opin Immunol 1996;8:878-85.
45. Rahyaman SM, Chira P, Koff RS. Idiopathic autoimmune chronic hepatitis triggered by Hepatitis A. Am J Gastroenterol 1994;89:106-8.
46. Robens JF, Richard JL. Aflatoxins in animal and human health. Rev Environ Contam Toxicol 1992;127:69-94.
47. Coulombe RA. Biological actions of mycotoxins. J Dairy Sci 1993;76:880-91.
48. Pestka JJ, Bondy GS. Immunologic effects of mycotoxins. In: Miller JD, Trenholm HL, editors. Mycotoxins in grain. St Paul (MN): Eagan Press; 1994. pp. 339-58.
49. Jackson LS, De Vries JW, Bullerman LB, editors. Fumonisins in food. New York: Plenum Press; 1996.
50. Neal GE. Genetic implication in the metabolism and toxicity of mycotoxins. Toxicol Lett 1995;82-83:861-7.
51. Creppy EE, Baudrimont I, Betbeder AM. Prevention of nephrotoxicity of ochratoxin A, a food contaminant. Toxicol Lett 1995;83-83:869-77.
52. Archer DL. Foodborne gram-negative bacteria and atherosclerosis: is there a connection? Journal of Food Protection 1987;50:783-7.
53. Farr SB, Kogoma Y. Oxidative stress response in *Escherichia coli* and *Salmonella typhimurium*. Microbiol Rev 1991;55:561-85.
54. Bruckner DA. Amebiasis. Clin Rev Microbiol 1992;5:356-69.
55. Wolfe MS. Giardiasis. Clin Microbiol Rev 1992;5:93-100.
56. Chappell CL, Matson CC. Giardia antigen detection in patients with chronic gastrointestinal disturbances. J Fam Pract 1992;35:49-53.
57. Smith JL. *Cryptosporidium* and *Giardia* as agents of foodborne disease. Journal of Food Protection 1994;56:451-61.

58. Keusch GT, Hamer D, Joe A, Kelley M, Griffiths J, Ward H. *Cryptosporidia*—who is at risk. Schweiz Med Wochenschr 1995;125:899-908.
59. Owen RJ. Bacteriology of *Helicobacter pylori*. Bailliere's Clin Microbiol 1995;9:415-46.
60. Fox JG. Non-human reservoirs of *Helicobacter pylori*. Aliment Pharmacol Ther 1995;9(Suppl 2):93-103.
61. Kuipers EJ, Uyterlinde AM, Pena AS, Roosendaal R, Pals G, Nelis GF, et al. Long term sequelae of *Helicobacter pylori* gastritis. Lancet 1995;345:1525-8.
62. Sipponen P, Kekki M, Seppala K, Siurala M. The relationship between chronic gastritis and gastric secretion. Aliment Pharmacol Ther 1996;10(Suppl 1):103-18.
63. Flegr J, Hrdy I. Influence of chronic toxoplasmosis on some human personality factors. Folia Parasitol (Praha) 1994;41:122-6.
64. Flegr J, Zitkova S, Kodym P, Frynta D. Induction of changes in human behaviour by the parasitic protozoan *Toxoplasma gondii*. Parasitology 1996;113:49-54.
65. Latman NS, Walls R. Personality and stress: an exploratory comparison of rheumatoid arthritis and osteoarthritis. Arch Phys Med Rehabil 1996;77:796-800.
